# Draft Genome Sequence of *Fusobacterium nucleatum* subsp. *nucleatum* ChDC F316, Isolated from a Human Peri-implantitis Lesion in the Republic of Korea

**DOI:** 10.1128/genomeA.01041-13

**Published:** 2013-12-12

**Authors:** Soon-Nang Park, Eugene Cho, Hwa-Sook Kim, Dae-Soo Kim, Jaeeun Jung, Jeong-Hun Baek, Yun Kyong Lim, Eojin Jo, Young-Hyo Chang, Jeong Hwan Shin, Sang-Haeng Choi, Jihee Kang, YongUn Choi, Hong-Seog Park, Hongik Kim, Joong-Ki Kook

**Affiliations:** Korean Collection for Oral Microbiology and Department of Oral Biochemistry, School of Dentistry, Chosun University, Gwangju, Republic of Koreaa; Department of Dental Hygiene, Chunnam Techno University, Chunnam, Republic of Koreab; Human Derived Material Center, Korea Research Institute of Bioscience and Biotechnology (KRIBB), Daejeon, Republic of Koreac; Macrogen, Inc., Gasan-dong, Seoul, Republic of Koread; Korean Collection for Type Cultures, Biological Resource Center, KRIBB, Daejeon, Republic of Koreae; Department of Laboratory Medicine, Busan Paik Hospital, Inje University College of Medicine, Busan, Republic of Koreaf; Research Institute of Unitech Science Co., Ltd., Daejeon, Republic of Koreag; R&D Division, Vitabio Inc., Daejeon, Republic of Koreah

## Abstract

*Fusobacterium nucleatum* is a Gram-negative anaerobe and is one of the causative agents of periodontal diseases, including peri-implantitis. *Fusobacterium nucleatum* subsp. *nucleatum* ChDC F316 (KCOM 1322) was isolated from a human peri-implantitis lesion. Here, we report the draft genome sequence of this strain.

## GENOME ANNOUNCEMENT

*Fusobacterium nucleatum* is a Gram-negative anaerobe and is one of the causative agents of periodontal diseases, including peri-implantitis (1–3). *F. nucleatum* was classified as five subspecies, *nucleatum*, *polymorphum*, *vincentii*, *fusiforme*, and *animalis*, based on the polyacrylamide gel electrophoresis pattern of the whole-cell proteins and DNA homology (4), 2-oxoglutarate reductase and glutamate dehydrogenase electrophoretic patterns, and DNA-DNA hybridization patterns (5). *F. nucleatum* subsp. *nucleatum* ChDC F316 (KCOM 1322) was isolated from a human peri-implantitis lesion. In this report, we present the draft genome sequence of *F. nucleatum* ChDC F316.

Draft sequencing was performed by the Macrogen Co., (Seoul, South Korea) using the Illumina Hiseq 2000 system sequencing technology. We constructed 101 paired-end sequencing libraries with insert sizes of about 200 bp and generated 35,690,994 bp of usable sequence. We assembled the reads using SOAPdenovo (http://soap.genomics.org.cn). SOAPdenovo v. 1.05 was run with option K79 and configuration options reverse_seq of 0 (standard mate-pair orientation), asm_flags of 3 (try harder to build large contigs), rank of 1 (reads were used while scaffolding). The reads were assembled into 83 contigs with a size range between 202 and 22,979 bp (total 2,162,122 bp), and the GC content was 26.95%. Open reading frames were predicted and annotated using the Glimmer-3.02 modeling software package (6). The predicted protein sequences were annotated as Gene Ontology (GO) by the basic local alignment search tool (BLAST). Then, the GO classes were grouped into a total of 124 GO-Slim terms using the web tool CateGOrizer (7).

The genome contained 2,077 protein-coding genes, three copies of 5S rRNA and 323 tRNA genes, and several key pathways for amino acids, carbohydrates, lipids, and organic acids. Biosynthetic pathways exist for four amino acids: glutamate, glutamine, aspartate, and asparagine. The strain might biosynthesized at least one more amino acid (glutamine) than the type strain (ATCC 25586) of *F. nucleatum* subsp. *nucleatum* (3). The draft genome sequence also contains virulence factors such as butyrate fermentation-related genes, hemolysin, zinc metalloprotease, serine protease, 5-nitroimidazole antibiotic resistance proteins, beta-lactamase, multidrug resistance proteins, multi-antimicrobial extrusion proteins, macrolide-efflux protein, outer membrane porin F, TonB protein, and tolC. The genome also contained oxidative stress-response genes such as glutathione peroxidase, glutaredoxin, NADH oxidase, and rubrerythrin (confers superoxide dismutase-like activity). The genome contained four two-component systems and one unmatched sensor histidine kinase.

### Nucleotide sequence accession numbers.

This whole-genome shotgun project has been deposited at DDBJ/EMBL/GenBank under the accession number ATKC00000000. The version described in this paper is the first version, ATKC01000000.
